# Reduced dispersal at nonexpanding range margins: A matter of disperser identity

**DOI:** 10.1002/ece3.6220

**Published:** 2020-04-16

**Authors:** Gilad Ben Zvi, Merav Seifan, Itamar Giladi

**Affiliations:** ^1^ Mitrani Department of Desert Ecology Swiss Institute for Dryland Environmental and Energy Research Jacob Blaustein Institutes for Desert Research Ben‐Gurion University of the Negev Midreshet Ben‐Gurion Israel

**Keywords:** dispersal, dispersal effectiveness, mutualism, myrmecochory, range margins, *Sternbergia clusiana*

## Abstract

The evolution of dispersal at range margins received much attention recently, especially in the context of dynamic range shifts, such as those following climate change. However, much less attention has been devoted to study variation in and selection on dispersal at nonexpanding range margins, where populations are often small and isolated, and empirical test is dearly missing. To fill this gap, we tested whether dispersal of an ant‐dispersed perennial plant (*Sternbergia clusiana*) is quantitatively and/or qualitatively reduced toward a nonexpanding range margin. We evaluated plant investment in dispersal structures (elaiosome), seed removal rates, and the relative abundance, activity, and behavior of low‐ and high‐quality seed‐dispersing ants in six sites ranging from mesic Mediterranean site to arid site (>600 to <100 mm of annual rainfall, respectively), which marks the southern range margin of the species. In a set of cafeteria and baiting experiments, we found that overall seed removal rates, the contribution of high‐quality dispersers, maximum dispersal distance and dispersal‐conducive ant behavior decreased toward range margins. These findings agree with a lower investment in reward by range margin plant populations, as reflected by lower elaiosome/seed ratio, but not by variation in the reward chemistry. More than variation in traits controlled by the plants, the variation in ant–seed interactions could be attributed to reduced presence and activity of the more efficient seed‐dispersing ants in the marginal populations. Specifically, we found a mismatch between local distribution of potentially effective seed dispersers and that of the plant, even though those dispersers were observed in the study site. Interestingly, although the observed variation in the outcome of ant–seed interactions supported the prediction of reduced dispersal at nonexpanding range margins with small and isolated populations, the underlying mechanism seems to be incidental difference in the seed‐dispersing ant community rather than a plant‐mediated response to selection.

## Graphical abstract

In this work, we show that the investment in a reward by a plant and the associated outcome of seed dispersal by ant declines towards a species range margin. However, the change in the outcome was mainly due to differences in the ant community that interact with seeds and less so by changes in the ant behavior.

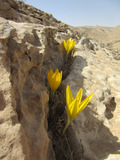

## 1 INTRODUCTION

Dispersal is a fundamental life history transition (Clobert, Danchin, Dhondt, & Nichols, 2001; Ronce, 2007) with numerous and significant ecological and evolutionary consequences (Clobert, 2012; Levin, Muller‐Landau, Nathan, & Chave, 2003; Nathan & Muller‐landau, 2000). Thus, dispersal‐linked traits are under considerable selection pressure (Bonte et al., 2012; Gandon & Rousset, 1999; Levin & Muller‐Landau, 2000). While the interspecific variations in dispersal syndromes and traits are often very noticeable, intraspecific variation may also play a major role in species’ success, especially along geographic and environmental gradients (Herrera, 2017; Manzaneda & Rey, 2008; Zelikova, Dunn, & Sanders, 2008). Intraspecific variation is of particular interest at a species range margin, where it may be most vulnerable to environmental changes due to anthropogenic and climatic factors (Hargreaves, Bailey, & Laird, 2015). In the context of range margins, dispersal is considered advantageous when the distribution of suitable habitats is not static. Dynamic shifts in the distribution of suitable habitat occur at a leading front of expanding populations, such as those following climate change (Travis et al., 2013). Dispersal will also be favored in nonexpanding range margins, as long as there is a high turnover in habitat suitability, which drives a metapopulation‐like dynamics involving local extinctions and colonization (Holt, 2003; Kubisch, Hovestadt, & Poethke, 2010; Travis & Dytham, 1999). However, when habitat turnover is low, populations at nonexpanding range margins, which often occupy small and isolated patches, will experience selection for reduced dispersal due to high probability of arriving at hostile matrix (Dytham, 2009; Hargreaves & Eckert, 2014). This prediction has yet to be tested by empirical studies, which lag far behind theoretical developments and predictions (Hargreaves & Eckert, 2014). This knowledge gap is especially serious for animal‐dispersed plants, where numerous studies focused on dispersal interactions in the context of dynamic range margins (Richardson, Allsopp, D'Antonio, Milton, & Rejmánek, 2000; Svenning et al., 2014), but studies concerning variation in animal‐dispersed plants at nonexpanding range margins are scarce.

For animal‐dispersed plants, dispersal and the selection forces that shape it may substantially depend on the distribution, behavior, and activity of potential seed dispersers, all of which may be sensitive to environmental conditions that sharply vary near range margins (Aslan et al., 2019). A conceptually useful approach to address this issue is to consider seed dispersal effectiveness as composed of the frequency of seed removal by each disperser (interaction quantity) and the outcome of each dispersal event in terms of plant recruitment (interaction quality; Schupp, Jordano, & Gómez, 2017). While evaluating the quantitative component across space, time, and species is relatively easy to accomplish, evaluation of the qualitative component is very challenging, as it is seldom feasible to follow seed fate from dispersal to recruitment in natural settings (Aslan et al., 2019). Nevertheless, in many zoochorous systems, the relative abundance, activity, and behavior of different seed disperses, which differ in the quality and quantity of dispersal service they convey to the plant (Azcárate & Manzano, 2011), can be used as a proxy for dispersal effectiveness (Schupp, Jordano, & Gómez, 2010). We tested whether an animal‐dispersed plant exhibits reduced dispersal toward its nonexpanding range margin. Furthermore, we evaluated whether such reduction, if it exists, is manifested in key quantitative and/or qualitative components of the seed dispersal effectiveness.

Myrmecochory, seed dispersal by ants, is a common dispersal syndrome across wide geographic ranges, ecosystem types, and plant taxonomic groups (Lengyel, Gove, Latimer, Majer, & Dunn, 2009, 2010). It is characterized by the production of an elaiosome, a lipid‐rich seed appendage that attracts certain groups of ant species and elicits seed carrying behavior (Giladi, 2006; Rico‐Gray & Oliveira, 2007). The key dispersers of myrmecochorous species are typically large scavenging ants (Gove, Majer, & Dunn, 2007; Hughes & Westoby, 1992b; Warren & Giladi, 2014). These ants are specifically attracted to the elaiosomes, which chemically mimic the scavengers’ natural insect diet (Pfeiffer, Huttenlocher, & Ayasse, 2010). Scavenging ants have high affinity to myrmecochoreous seeds and are considered effective seed dispersers because they transport the seeds over long distances, consume the elaiosome only, and leave the seeds intact outside their nest, affecting positively the plants demographic performance (Gorb, Gorb, & Punttila, 2000; Hanzawa, Beattie, & Culver, 1988). In comparison, other ant guilds, such as granivorous ants, show low affinity to the myrmecochore seeds and provide inferior dispersal services both quantitatively and qualitatively, often consuming the seeds after their removal (Hughes & Westoby, 1992a, 1992b). The plant's seed dispersal effectiveness may thus be estimated by weighing seed removal rates by various ant guilds, while considering the relative quality of the seed dispersal services provided by each guild.

We tested whether dispersal characteristics of a myrmecochorous species with long‐persisting populations in well‐defined habitats, and which mainly interacts with two ant guilds, vary toward its southern range margin. Specifically, we predict that due to the hypothesized high dispersal cost at such nonexpanding range margins, localized dispersal will be favored. We further predict that the reduction in dispersal may originate from two, nonmutually exclusive, processes: First, plant‐mediated reduction in seed dispersal effectiveness may be related to lower elaiosome attractiveness at range margins. In general, the probability of a seed to be dispersed by a high‐quality disperser increases with elaiosome size or elaiosome/seed mass ratio (Edwards, Dunlop, & Rodgerson, 2006; Levine, Ben‐Zvi, Seifan, & Giladi, 2019; Mark & Olesen, 1996) and with the inclusion of higher concentrations of specific fatty acids (most prominently oleic acid) in the elaiosome content (Boulay, Coll‐Toledano, & Cerdá, 2006; Mayer, Ӧlzant, & Fischer, 2005). We hypothesized that plants in range margin populations, which inhabit small patches of suitable habitat, will produce smaller and less attractive elaiosomes, thus securing more localized dispersal than populations closer to the range core. Second, ant‐mediated seed dispersal at range margins may be limited by the absences and or low abundance of high‐quality seed‐dispersing ants. This is expected if similar environmental conditions, such as an increase in aridity, limit in a similar way the distribution of both plants and their efficient seed dispersers. In particular, we predict that efficient seed dispersers may be replaced at range margins by other less efficient species (see Manzaneda, Rey, & Boulay, 2007; Zelikova et al., 2008 for variation in disperser assemblage across a plant's range). In this scenario, dispersal effectiveness may be lower at range margins regardless of variation in the diaspore traits.

## 2 METHODS

### 2.1 Study species and study sites

*Sternbergia clusiana* (*Amaryllidaceae*) is a bulbous herbaceous perennial myrmecochore, which flowers in the autumn, sets leaves with the onset of rains (December–March), and produces mature fruits (1–6 cm long and 1–3 cm in diameter) in March–April (Boeken & Gutterman, 1989). Each fruit contains 5–200 seeds (27.6 ± 20.9 $\bar{X}\pm SD$ ), each equipped with a large elaiosome to form a large (109.6 ± 40.5 mg) diaspore. The species distribution covers the eastern Mediterranean region (Livneh & Heller, 1986; Mathew, 1983, 1984; Shmida & Fragman‐Sapir, 2015 Youssef, Mahmood, & Vela, 2017). Within the study region, *S. clusiana* is found in ca. 60 sites that are distributed along a sharp precipitation gradient ranging from Mediterranean climate in the north (>600 mm of annual rainfall) to arid climate (<100 mm of annual rainfall) in the Negev desert, which marks the southern range margin of the species (Shmida & Fragman‐Sapir, 2015, Figure 1). The populations within the vast majority of these sites are confined locally to specific well‐defined habitat type and are of limited extent, thus forming clearly isolated populations. For this study, we chose six *S. clusiana* populations representing the geographical distribution of the species toward its southernmost distribution margins (Table 1).

**FIGURE 1:**
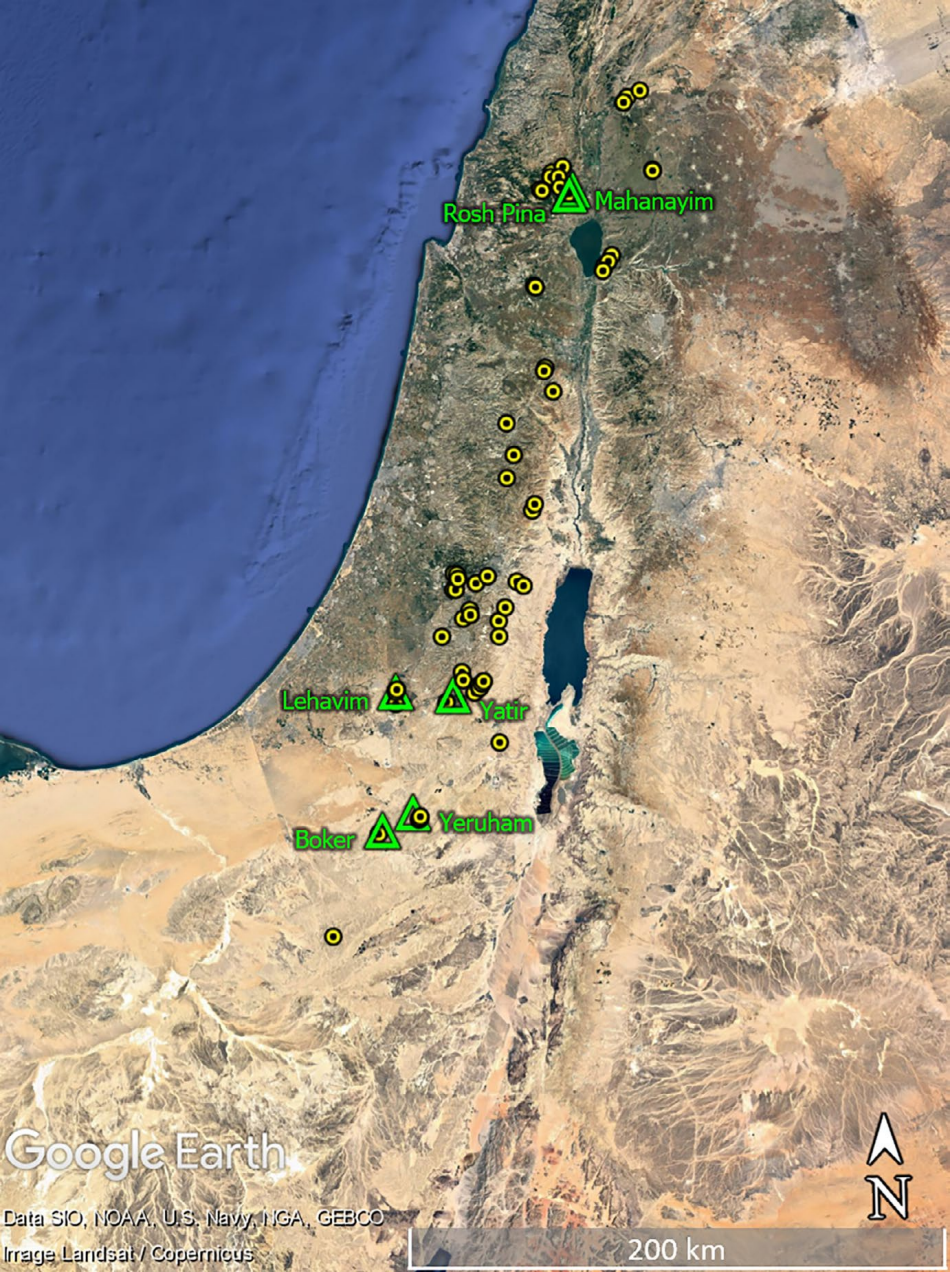
Distribution map of Sternbergia clusiana populations within the study regions (yellow circles) and the location of the populations that were sampled (green triangles)

**TABLE 1:** Selected study sites

Site	Region	Location (long.–lat.)	Altitude (m A.S.L)	Annual rainfall (mm, 1981–2010)	Average temp. (°C, January/July, 1995–2009)	Lithology	Distance in km from southernmost population	Aspect and slope
Wadi Mahanayim	Upper Galilee	35°32′01″E 32°59′25″N	550	<671	8.9–25.3	Eocene chalk and limestone	287	Steep south‐facing slopes
Wadi Rosh Pina	Upper Galilee	35°31′12″E 32°58′31″N	750	<671	8.1–25.9	Eocene chalk and limestone	285	Steep south‐facing slopes
Yatir Forest	Northern Negev	35°02′58″E 31°21′17″N	650	271	9.7–25.3	Turonian chalk and limestone	93	Various aspects and slopes
Lehavim	Northern Negev	34°49′42″E 31°22′04″N	350	297	12.1–27.8	Eocene chalk + Quaternary Loess	86	Various aspects and slopes
Yeruham	Central Negev	34°53′16″E 30°58′27″N	530	98	9.7–26.2	Cenomanian limestone	61	North‐facing rocky outcrops
Boker Ridge	Central Negev	34°46′15″E 30°54′59″N	490	93	9.9–26.2	Turonian limestone	51	North‐facing rocky outcrops

Note

Climate data retrieved from Israel Meteorological Service (2018). Lithology data retrieved from Sneh, Bartov, Weissbrod, and Rosensaft (1998).

Within the study region, two ant genera, *Messor* and *Cataglyphis*, are mostly involved in handling and carrying seeds of myrmecochore species, including *S. clusiana* (Galil, 1952; Danin & Yom‐Tov, 1990; Table 1.1.1 in Appendix S1). Of these, three granivore species (*Messor ebeninus*, *M. semirufus,* and *M. arenarius*) and three scavenger species (*Cataglyphis savignyi*, *C. israelensis,* and *C. albicans*; Eyer, Seltzer, Reiner‐Brodetzki, & Hefetz, 2017; Vonshak & Ionescu‐Hirsch, 2009) are the most common around *S. clusiana* sites.

### 2.2 Characteristics of *S. clusiana* seed dispersal toward range margin

Characteristics of seed dispersal by ants were evaluated in 24 cafeteria experiments that were conducted in each of the 6 study sites (a total of 144 experiments). In each site, we identified 6 nests of each of the two ant guilds (scavengers and granivores), and we placed one seed depot, consisting of 15 freshly collected *S. clusiana* seeds, near (1 m) and a second depot far (10 m) from the opening of each of these focal nests. The exact direction of the seed depots, while randomly selected for the scavenger *Cataglyphis*, was dictated by active foraging trails in the case of the granivorous *Messor* ants, which would otherwise fail to detect the depots within a reasonable experimental time. Half of the nests that were used for these experiments were at the center of the local *S. clusiana* population (high *S. clusiana* density) and half next to the population boundary (low density). Due to a very narrow and highly aggregated distribution of *S. clusiana* in the two range margin sites (Boker and Yeruham), the population “boundary” depots were located 20–40 m outside the actual population boundary.

In each experiment, we evaluated dispersal quantity by estimating the removal rate of *S. clusiana* seeds. As proxies for dispersal quality, we calculated the proportion of seeds removed by each ant guild and by recorded seed removal distances. We monitored each seed depot for 90 min. Whenever an ant interacted with a seed we recorded: (a) ant species; (b) dispersal distance, in cases where a seed was removed (regardless of the destiny of the seed). In addition, we recorded ant–seed interaction index, following Culver and Beattie (1978), as an auxiliary variable, which represents the interaction strength categorized by five ranks: ignoring (1), antennae contact (2), mandible contact (3), pickup/ failed removal (4), and successful removal (5; see further details in Table 1.2.1 in Appendix S1). Using these observations, we calculated for each seed depot (a) the probability of seeds to be removed from a depot; (b) scavenger/granivore seed removal ratio, and (c) averaged value of the interaction index for each ant guild.

### 2.3 Assessment of variation in *S. clusiana's* diaspore traits

In order to test whether variation in diaspore traits can explain variation in seed dispersal, we collected hundred ripe diaspores from 20 *S. clusiana* individuals in each site, weighted the elaiosome and the seed and calculated the elaiosome/seed mass ratio as an assessment of the relative investment in ant attractants (see Edwards et al., 2006; Hughes & Westoby, 1992b). We also tested for potential chemical variation in elaiosome content across sites. We analyzed elaiosomes from 10 *S. clusiana* individuals from each site (minimum 20 mg total dry mass per individual, collected from 4 elaiosomes per sample). Using Pal, Khozin‐Goldberg, Cohen, and Boussiba (2011) protocol, we extracted fatty acids and constructed their profile and composition (for full procedure see Appendix S1.3). The results were compared across sites. We specifically focused on the amount of oleic acid which is known to elicit seed removal behavior by ants (Mayer et al., 2005).

### 2.4 Assessment of ant presence and activity among sites

In order to evaluate whether the outcome of the cafeteria experiments is affected by ants’ presence and activity, we used baits consisting of tuna chunks and a few hundred quinoa seeds to attract both scavengers and granivores (Agosti, Majer, Alonso, & Schultz, 2000; Ryder Wilkie, Mertl, & Traniello, 2010). Although this approach does not necessary provide a comprehensive assessment of the whole ant community in a site, it targets (and may even over‐represent) those ant species that are most relevant to seed dispersal (Warren & Giladi, 2014). The baits were placed immediately after each cafeteria experiment along transects connecting the nest opening and the “Far” depot at distances of 0, 2.5, 5, 7.5, and 10 m from each nest opening, and then monitored for 60 min at 10‐min intervals during which we determined the identity and number of ants visiting each bait.

### 2.5 Statistical analysis

We tested the effects of site, ant guild (i.e., the guild inhabiting the nearest nest to the seed depot), distance from that nest (distance), and within‐site location of depot in relation to *S. clusiana* density (center versus. boundary, termed “location”) on the probability of seeds removed from a depot. The data set included many observation sessions summed to either all or none of the seeds being removed from a depot. Due to this unique, bimodal nature of the data set, we used a mixture model with beta‐binomial error distribution following a procedure used by Bolker (2008). We first selected the most appropriate error distributions for the seed removal data, after considering all combinations of zero‐inflated and non‐zero‐inflated binomial and beta‐binomial distributions. Second, once the beta‐binomial distribution was selected, we explored optional parametrizations of the beta‐binomial model, where each parameter was either held constant or could vary as a function of the explanatory variables (see Appendix S1.4 for detailed description of the model construction). Last, we used a beta‐binomial model with the selected parametrization to run generalized linear models. This set included all possible combinations of the effects of the four explanatory variables mentioned above and their 2‐way interactions. We then used ΔAIC_c_ to select the most parsimonious model.

We further analyzed the potential effects of the same four factors (site, ant guild, distance to the nearest nest, and within‐site location) and their 2‐way interactions on averaged values of the ant–seed interaction index per seed depot. We selected the best model of all combinations of these effects using ΔAICc. The nature of the data set dictated the usage of GLMs with normal distribution (log link). We then inferred the particular effects of each explanatory variable in the best‐fitting model using Wald chi‐square tests.

As disperser identity may affect dispersal quality, we evaluated for each seed depot the ant guilds’ seed removal ratio, that is, the number of seeds removed by scavengers divided by the number of seeds removed by granivores calculated as: Log[(scavenger + 1)/(granivore + 1)]. The statistical analysis procedure followed the same order as that of the interaction index, but the nature of the data set required the usage of GLM with gamma distribution (log link). Finally, we examined another aspect of dispersal quality by comparing differences in dispersal distances in these experiments. As many of the seeds were either left in situ or taken to the nearest nest, the distribution of the dispersal distances was evidently biased by the distance of the depots from the ant nests (1 and 10 m), and we therefore only employed a qualitative comparison.

We tested for the potential trend in the investment in elaiosome toward *S. clusiana* range margin using GLMs (normal distribution) by including the effect of site on elaiosome to seed mass ratio (identity link), elaiosome total fatty acid content (log link), and the contents of specific fatty acids in the elaiosome (identity link). The potential effects of site, ant guild (i.e., the guild inhabiting the focal nest), distance from nest (distance), and within‐site location of the seed depot on the average number of ants visiting a bait were analyzed using a GLM (normal distribution with a log link). This analysis was conducted separately for the two ant guilds. The generalized linear mixture model with the beta‐binomial distribution was implemented using the R software 3.4.3 (R Development Core‐Team, 2017) and the following packages: emdbook (Bolker, 2020a) and bbmle (Bolker, 2020b). All other analyses were executed in SPSS 25 (IBM, 2017). All graphics were executed in R software 3.4.3 (R Development Core‐Team, 2017).

## 3 RESULTS

### 3.1 Assessment of *S. clusiana* seed dispersal toward range margin

**FIGURE 2:**
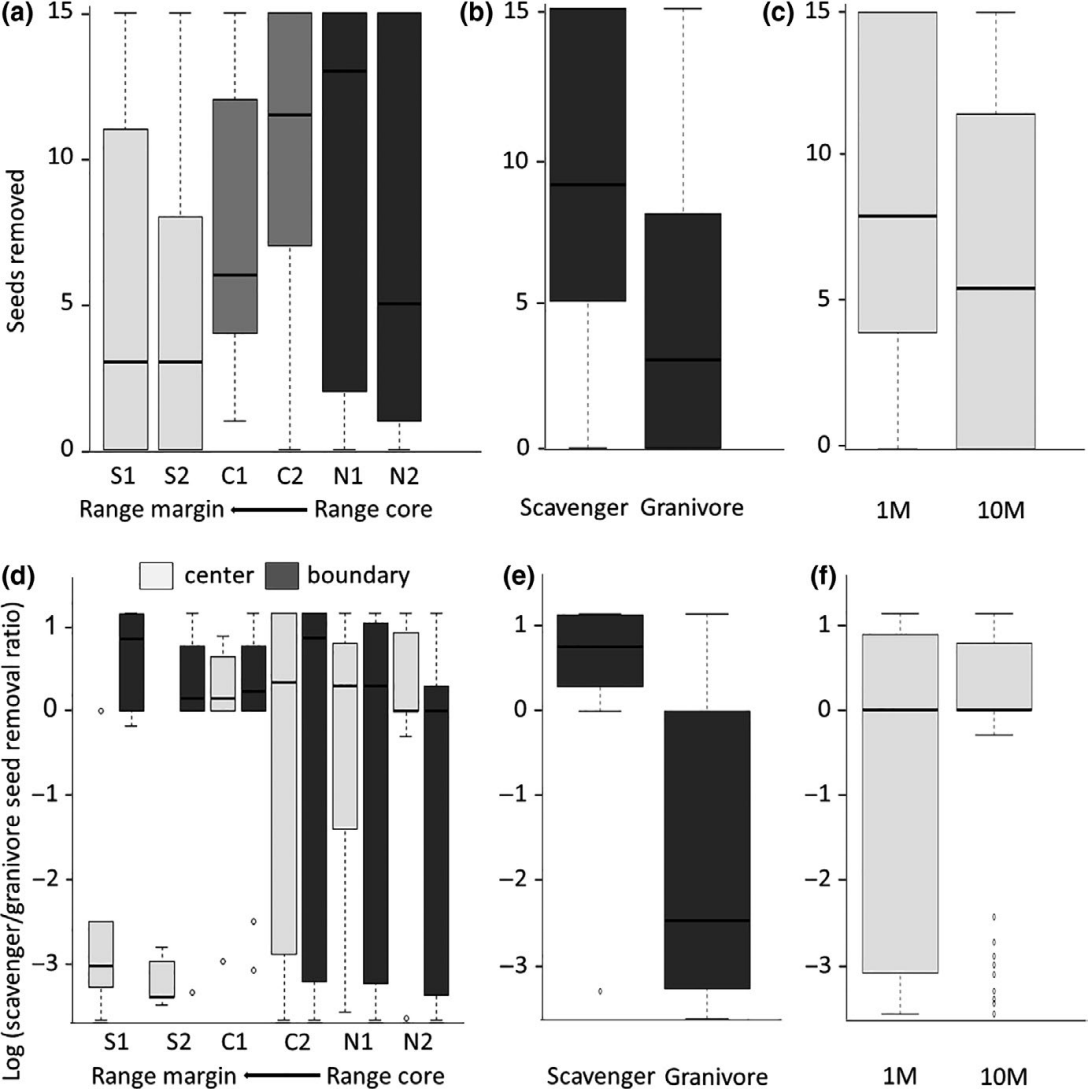
Seed removal rates across populations (a), the identity of the ants occupying the nest to which seed depot was presented; (b) and depot's distances from nests (c); and the ratio of seeds removed by scavengers relative to seeds removed by granivores (log transformed) in each seed depot across populations and locations within populations (d), across ant guilds (e), and across depot's distance from nest (f). S1, S2—Central Negev populations, representing the range margin; C1, C2—Northern Negev; N1, N2 Upper Galilee populaitons. Each boxplot represents the median (black horizontal line), 25 and 75 quartiles. Error lines represent the maximal and minimal results, unless there are suspected outliers (unfilled circles): In this case, error bars represent the “inner fence” (1.5× likely range of variation from the quartile)

The most parsimonious model that explains seed removal rate included the effects of site, the ant guild occupying the focal nest, and distance of the depot from the nest, but no interaction terms (see Tables 2.1.1–2.1.3 in Appendix S2). Particularly, we found that the probability of *S. clusiana* seeds to be removed by ants decreased in sites located at the species’ range margin ( $\chi_{5}^{2}=22.09,p<.001$ , Figure 2a, Tables 2.1.2–2.1.3 in Appendix S2). In addition, removal probabilities were higher at seed depots associated with scavenging ant nests ( $\chi_{1}^{2}=19.45,p<.001$ , Figure 2b, Tables 2.1.2–2.1.3 in Appendix S2) and when depots were located closer to the nest ( $\chi_{1}^{2}=9.14,p=.003$ ), Figure 2c, Tables 2.1.2–2.1.3 in Appendix S2). The guild of the ants visiting a depot was frequently identical to the guild occupying the adjacent focal nest, especially as the granivores visited almost exclusively the seed depots near their own nests.

The ratio of seeds removed by scavenger versus granivore ants was best explained by a model that included the effects of site ( $\chi_{5}^{2}=23.91,p<.001$ , Figure 2d), within‐site location ( $\chi_{1}^{2}=11.83,p=.001$ , Figure 2d), focal nest guild ( $\chi_{1}^{2}=28.00,p<.001,$ Figure 2e), depot's distance from nest ( $\chi_{1}^{2}=7.96,p<.005$ , Figure 2f), and the following interactions: site × location ( $\chi_{5}^{2}=42.47,p<.001$ , Figure 2d), site × focal nest guild ( $\chi_{5}^{2}=20.67,p=.001$ , Figure 2.1 in Appendix S2), and location × distance ( $\chi_{1}^{2}=11.80,p=0.001$ , Figure 2.1 in Appendix S2; full information on model selection is found in Table 2.2.1 in Appendix S2). In particular, the interaction between site and within‐site location reflects the observation that at *S. clusiana's* range margin, the relative contribution of scavenging ants to seed removal at the center of *S. clusiana* local populations was much lower than at the boundary of the population (Figure 2d, Table 2.2.2 in Appendix S2).

**FIGURE 3:**
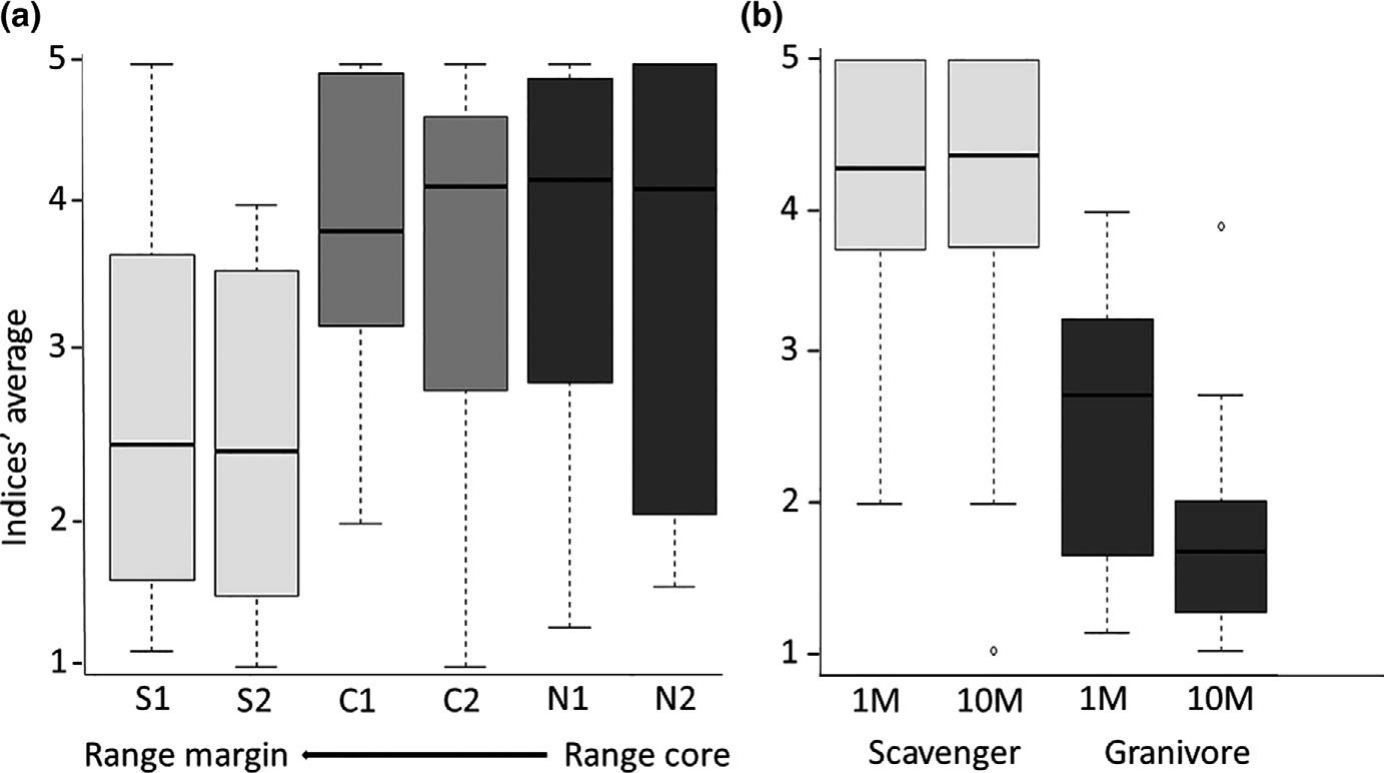
Average values of the interaction indices per seed depot across populations (a) and across ant guild's nest and depot's distance from the nest (b). Boxplots represent the median (black horizontal line), 25 and 75 quartiles. Error lines as described in Figure 2

**FIGURE 4:**
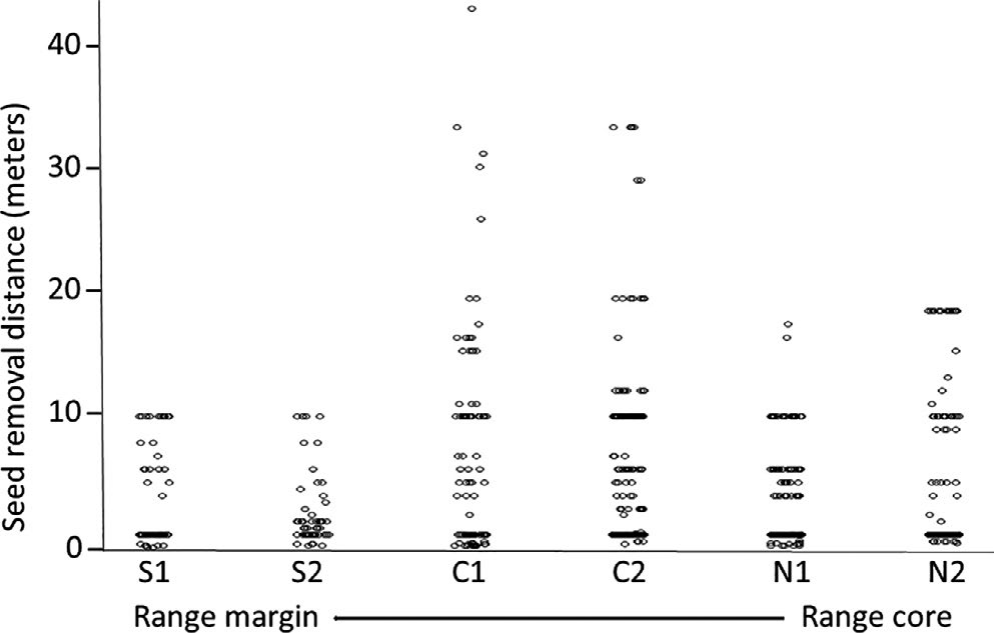
Histogram of seed removal distances across populations. Each dot represents a single seed displacement distance

Variation in the average ant–seed interaction index per depot was best explained by a model that included site ( $\chi_{5}^{2}=58.75,p<.001$ ), focal nest guild ( $\chi_{1}^{2}=161.38,p<.001$ ), depot's distance from the focal nest ( $\chi_{1}^{2}=10.14,p=.001$ ), and the interaction between focal nest guild and distance ( $\chi_{1}^{2}=8.02,p=.005$ ; for full information on model selection see Table 2.2.1 in Appendix S2). Overall, ant–seed interaction tended to be less intense toward *S. clusiana* range margins (Figure 3a, Table 2.2.2 in Appendix S2). Moreover, Scavengers had higher interaction intensity than granivores, and unlike granivores, the scavenger's interaction intensity did not decrease with distance from the focal nest (Figure 3b; Table 2.2.2 in Appendix S2). In addition, the graphical inspection showed that overall seed removal distances were lower toward range margins (Figure 4).

### 3.2 Assessment of variation in *S. clusiana's* diaspore traits

**FIGURE 5:**
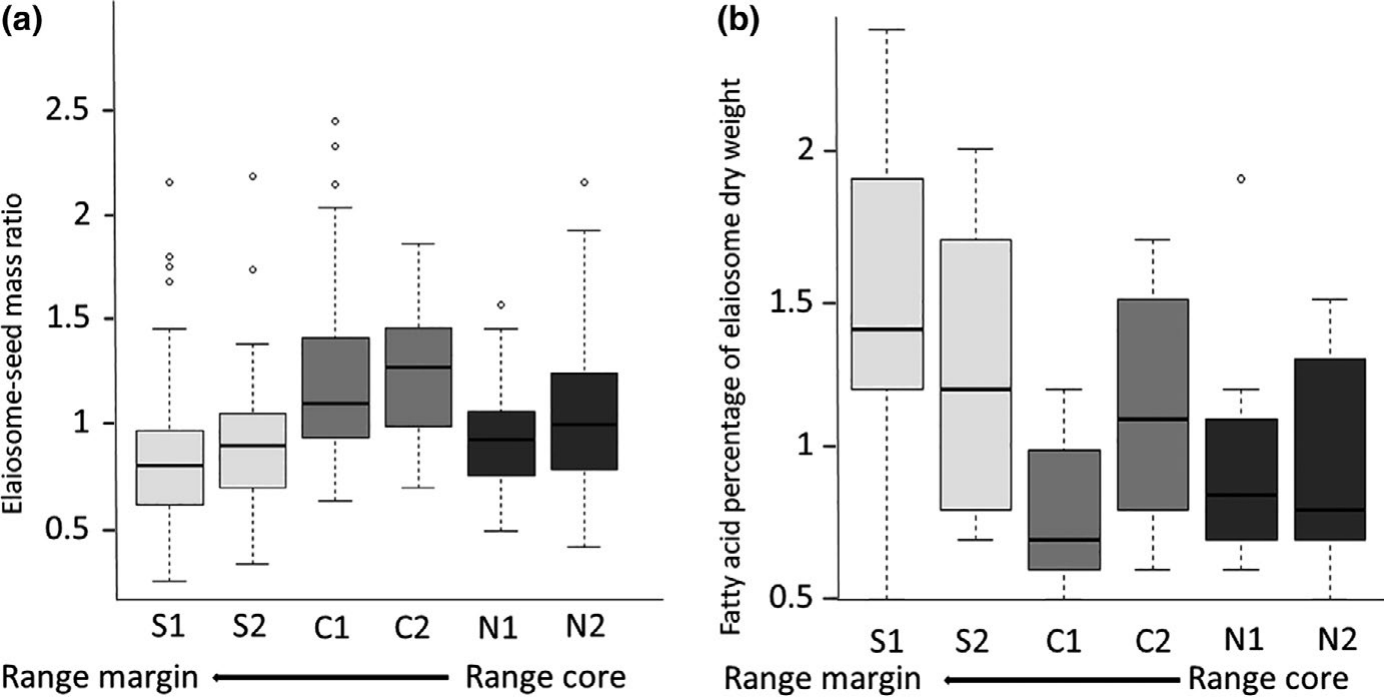
Elaiosome traits across populations: elaiosome/seed mass ratio (a); total fatty acid content (b). Boxplots represent median (black horizontal line), 25 and 75 quartiles. Error lines as described in Figure 2

Overall, eliaosome to seed mass ratio was lower in sites located at *S. clusiana*'s range margins ( $\chi_{5}^{2}=157.22,p<.001$ ; Figure 5a; Table 2.3.1 in Appendix S2). However, total fatty acid content of elaiosomes from range margin populations was higher than from populations away from the range margin ( $\chi_{5}^{2}=19.72,p=.001$ ; Figure 5b; Table 2.3.1 in Appendix S2). When we focused on oleic acid only, site effect was significant ( $\chi_{5}^{2}=22.58,p<.001$ ), mainly due to a very low oleic acid in one of the northern sites (Figure 2.3b, Table 2.3.1 in Appendix S2). Additionally, when the overall content of fatty acids was compared among sites, we could not detect a clear trend (Figure 2.3c in Appendix S2).

### 3.3 Assessment of ant presence and activity level

**FIGURE 6:**
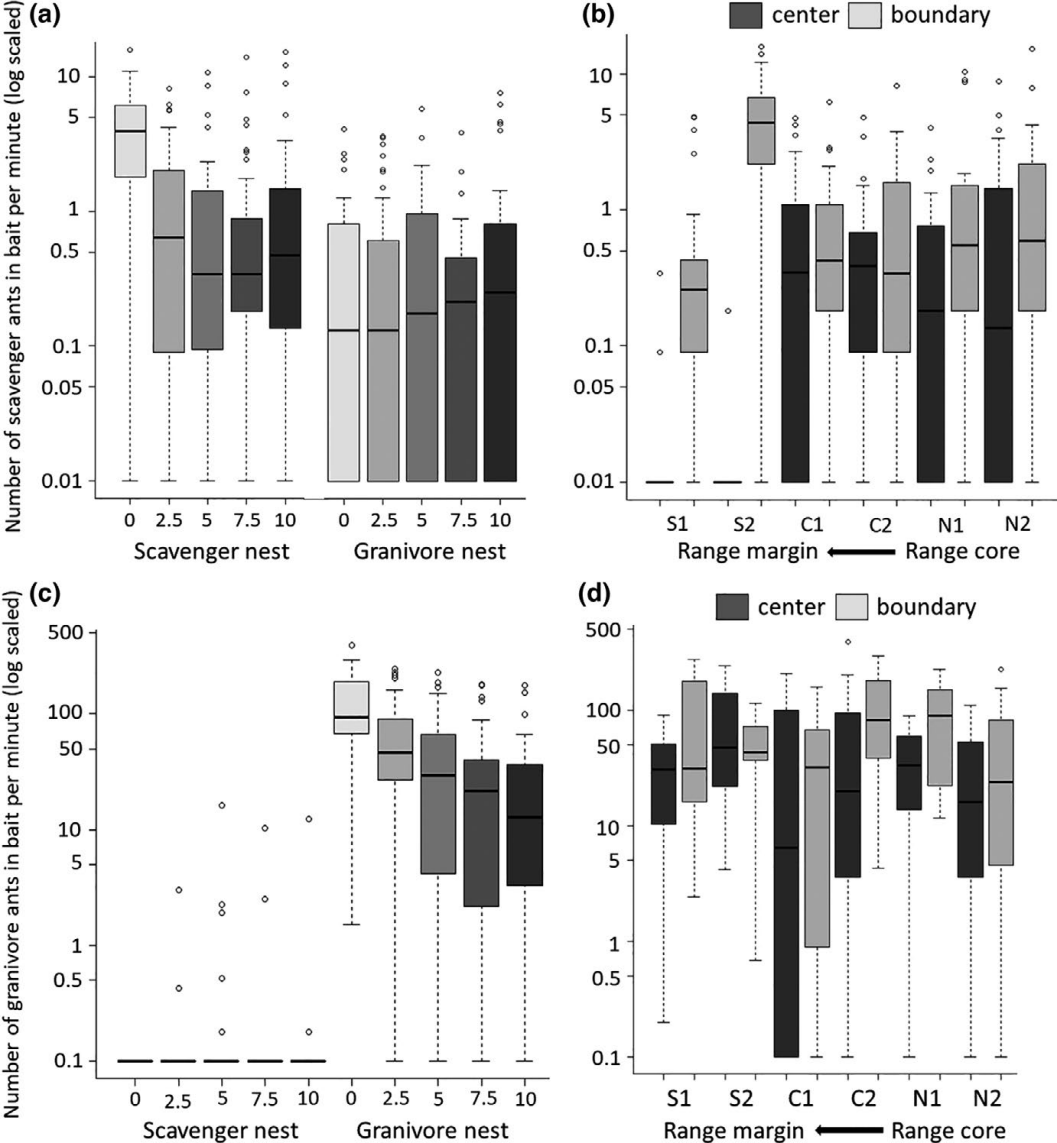
Results of the numbers of ants per minute in baits: (a) Scavenger ants across focal nest genera and baits’ distances from nest (0, 2.5, 5, 7.5, and 10 m). (b) Scavenger ants across sites and within‐site locations. (c) Granivore ants across focal nest genera and baits’ distances from nest. (d) Granivore ants (only near nests of granivores) across sites and within‐site locations. Boxplots represent median (black horizontal line), 25 and 75 quartiles. Error lines as described in Figure 2

By and large, the results from the bait experiment mirror the results from the cafeteria experiments and indicate that differences in seed removal by the two ant guilds are affected by spatial variation in the ants' presence and activity patterns. The number of scavenging ants attending baits was higher at baits located next to scavenging ant nests than baits located next to granivorous ants nests ( $\chi_{1}^{2}=25.89,p<.001$ ). The number of scavenging ants at baits seems to decrease with distance from a nest, but only with respect to scavenger nests (ant guild nest*distance, $\chi_{4}^{2}=18.16,p<.001$ ; Figure 6a). Scavenging ants arrived at most bait locations (275 out of 345 locations), with the exception of the centers of the range margin populations, where no nests of scavenging ants were found and where these ants rarely foraged (Figure 6b). On the other hand, their number was especially high in one range margin site ( $\text{Yeruham};\chi_{1}^{2}=5.36,p=.021$ ), Figure 6b; Tables 2.4.1–2.4.3 in Appendix S2). Granivorous ants exhibited a different spatial behavior. They were very active along transects that radiated from nests of granivorous ants, but were rarely observed next to nests of the scavenging ants (in only 10 out of 180 locations; see Figure 6c). Therefore, for granivorous ants we ran a generalized linear model only for baits near nests of granivores excluding the focal nest genus as a factor. The average number of granivorous ants attending the baits decreased significantly with distance from the nest entrance ( $\chi_{4}^{2}=60.67;p<.001$ ; Figure 6c), a pattern that remained consistent across sites and locations. Their number also varied among study sites ( $\chi_{5}^{2}=20.10,p=.002$ ), among locations within site, and was higher at populations boundary than at their center ( $\chi_{1}^{2}=5.21,p=.022$ ) and among the different combinations of site and location ( $\chi_{1}^{2}=11.82,p=.037$ ; Figure 6d; Tables 2.4.1–2.4.2, 2.4.4 in Appendix S2).

## 4 DISCUSSION

In this study, we showed that seed dispersal is generally reduced toward the range margin of a myrmecochore species. Moreover, we showed that the reduction in seed dispersal is not necessarily driven by variation in the level of the seed attractiveness to the ants, but by reduced availability of effective dispersal agents at the plant's range margin. Theory predicts a selection for limited dispersal at nonexpanding range margins whenever small size, isolation, and low temporal turnover of suitable habitat patches incur high cost of dispersal (Dytham, 2009; Hargreaves & Eckert, 2014). We suggest that the distribution and dynamics of *S. clusiana* populations in its southern range margin fit those characteristics of nonexpanding range margins that reflect selection for reduced dispersal. Particularly, populations of *S. clusiana* within the study region have been monitored for decades and have been repeatedly observed in the same well‐defined patches (Ivri, 1986). This is especially true for the southern populations (Livneh, 2016), which have high affinity to soil pockets at the base of rocky outcrops with improved edaphic conditions within an inhospitable arid environment (Boeken & Gutterman, 1989). Our finding contributes unique empirical evidence for the rarely tested theoretical prediction of lower dispersal at range margins with low temporal habitat turnover (Hargreaves & Eckert, 2014). To the best of our knowledge, the only other empirical study dealing with variation in dispersal potential in relation to nonexpanding range margins is by Darling, Samis, and Eckert (2008). In their study, they reported an increase in dispersal toward range margins, attributing it to high demographic instability at range margins.

Interestingly, there was no consistent trend in the variation in elaiosome traits with respect to the geographic gradient toward the range margin of *S. clusiana*. On one hand, range margin populations showed the lowest elaiosome‐seed mass ratios (see Figure 5), potentially indicating a relatively low investment in the reward that is associated with attracting scavenging ants (Edwards et al., 2006; Mark & Olesen, 1996). On the other hand, the elaiosome's chemical composition, and, in particular, the variation in oleic acid content, the fatty acid that elicits seed carrying behavior by ants (Boulay et al., 2006; Mayer et al., 2005), did not show a clear geographic trend in our study. Moreover, the elaiosome's total fatty acid content was highest in the range margin populations, indicating relatively high investment in reward. The elaiosome traits (i.e., mass and chemistry) that may mediate selection for reduced dispersal at range margins are those to which ants respond most. The decrease in the average ant–seed interaction index toward range margins suggests that elaiosome mass affects ant behavior more than elaiosome chemistry, but only detailed experimental work, in which elaiosome size and chemistry can be independently manipulated (e.g., by using of artificially manipulated elaiosomes), can provide a mechanistic explanation to this observation.

The variation in the composition and behavior of the ant community is consistent with our prediction of reduced dispersal of *S. clusiana* at range margin. This finding concurs with other studies dealing with the effects of partner distribution and community composition on dispersal in general (Willson & Traveset, 2000), and on myrmecochory in particular (Manzaneda & Rey, 2008; Zelikova et al., 2008). At the range margin sites, there was a mismatch between the local distribution of the scavenging ants, potentially the more effective seed dispersers, and that of *S. clusiana*, resulting in low quantity and apparent low quality of seed dispersal. This mismatch is fine‐scaled. Large scavenging ants are common and active in a close vicinity (~100 m) of *S. clusiana* populations, but rarely forage within the very microhabitats occupied by the plant. Such mismatches between dispersers and the distributions of myrmecochorous plants have also been found along elevation or soil moisture gradients (Warren, Giladi, & Bradford, 2010; Zelikova et al., 2008). Local‐scale mismatch may be typical for species at their range margins, where scarcity and patchiness of suitable habitat may push mutualistic partners to inhabit unique, yet distinct microhabitats (Lawson, Bennie, Hodgson, Thomas, & Wilson, 2014).

We propose two mechanisms that may underlie the observed mismatch between the local distributions of the plant and its presumably effective seed disperser at range margin populations. First, that mismatch may simply reflect a mismatch in the local distributions of environmental conditions that can support and/or being preferred by each partner. The north‐facing rocky outcrops to which *S. clusiana's* populations are virtually restricted at the arid range margin (Boeken & Gutterman, 1989), are relatively steep, shaded, and cool, and enjoy a profuse amount of runoff (Danin, 1999). This may make them less attractive to the thermophilic scavenger *Cataglyphis* (Wehner & Wehner, 2011). Second, in addition to habitat patches that are either suitable for the plant or for the ant, range margins also host patches that can potentially support both partners. However, in those patches where the environmental requirements of both partners intersect, dispersal by scavenging ants away from these patches incurs high dispersal cost and may lead to a local extinction of *S. clusiana*. Consequently, the only patches where sizable populations of *S. clusiana* can persist at the range margin are those where scavenging ants are locally absent (due to other reasons). Thus, the decrease in elaiosome/seed mass ratio, which indicates a lower attractiveness to otherwise high‐quality partners, may actually prove beneficial toward range margins, where lower dispersal rates and shorter distances are advantageous (Dytham, 2009; Hargreaves & Eckert, 2014).

The suggestion that a beneficial partner may become a burden under some circumstances necessitates a word of caution with respect to a common association of partner identity with dispersal quality. Clearly, without a direct and comprehensive measure of seed fate following dispersal by different partners, using disperser identity as a proxy for dispersal quality is problematic. More specifically, it is important to evaluate whether these proxies are transferable across systems and regions. Our preliminary data conducted in the vicinity of the range margin populations supported the working assumption that seeds of *S. clusiana* removed by scavenging ants experience drastically lower predation, higher re‐dispersal from the nest, and higher probability of arrival to potential safe sites than those dispersed by granivorous ants (G. Ben‐Zvi, unpublished). Still, to fully answer this question, we need a further study that encompasses the full treatment of seeds by each ant guild, including the postremoval stages of dispersal (Servigne & Detrain, 2010; Vander Wall & Longland, 2005) and the recruitment rates in both range margin and other populations.

At a wider context, the selection for decreased dispersal at range margin, which benefits the individual, may have deleterious effects at the population level, leading to increased aggregation and consequently intraspecific competition, and even to a failure to track changes in the distribution of suitable habitat (Gyllenberg, Parvinen, & Dieckmann, 2002; Urban, Tewksbury, & Sheldon, 2012). Climate change induces aridity and increasing temperatures in many regions including the Mediterranean. As the responses of species to climatic changes are seldom fully coordinated and synchronized, biotic interactions are predicted to experience profound changes (Berg et al., 2010). Consequently, temporal and spatial mismatches between mutualists are expected to increase in number and intensity (Rafferty, Caradonna, & Bronstein, 2015; Warren & Bradford, 2014). A spatial mismatch, in which an obligate mutualist population's distribution is asynchronized with its partner's distribution, conveys a long‐term extinction risk. Isolated range margin populations, which are sensitive to global change due to their lower adaptive potential (Bridle, Polechová, Kawata, & Butlin, 2010), are especially prone to similar spatial mismatches and consequently to a higher extinction risk (Gilman, Urban, Tewksbury, Gilchrist, & Holt, 2010). This risk may be alleviated by the replacement of the original mutualist with a new one, a role which in our study system granivorous ants may take in the southern range margin of *S. clusiana*. Moreover, the alternative partners, even if providing less effective services compared to the original ones, are potentially better adapted to higher temperatures and aridity, becoming more effective (Stuble et al., 2014). Even inferior mutualists may thus provide a buffer against climate change‐induced extinction. Understanding the extent to which new partnerships can efficiently replace old ones is an issue that is of great theoretical and applied interest and importance.

## CONFLICT OF INTERESTS

The authors have no competing interests to declare.

## AUTHOR CONTRIBUTION

**Gilad Ben Zvi:** Conceptualization (supporting); Formal analysis (lead); Investigation (lead); Methodology (equal); Visualization (lead); Writing‐original draft (lead); Writing‐review & editing (equal). **Merav Seifan:** Conceptualization (equal); Formal analysis (supporting); Funding acquisition (equal); Investigation (supporting); Supervision (equal); Writing‐original draft (supporting); Writing‐review & editing (equal). **Itamar Giladi:** Conceptualization (lead); Formal analysis (supporting); Funding acquisition (equal); Investigation (supporting); Project administration (lead); Writing‐original draft (supporting); Writing‐review & editing (equal). 

## Supporting information

Appendix S1

Appendix S2

## Data Availability

Data used for all the analyses and for the production of the graphical presentation included in this manuscript will be uploaded to the Dryad database https://doi.org/10.5061/dryad.612jm640j.
