# Supplementary material for: Reduced dispersal at nonexpanding range margins: A matter of disperser identity
Source: Ecol Evol. 2020 Apr 16;10(11):4665–76. doi: 10.1002/ece3.6220 (PMC7297755; doi:10.1002/ece3.6220)
Supplement: Supplementary file 1 — Appendix S1 [file ECE3-10-4665-s001.docx]

**Appendix S1 – methods complementary:**

**Table 1.1.1:** Ant species in the cafeteria experiment:

| **Ant species** | **Dietary guild** | **Worker body size (mm)** | **Foraging strategy** | **Reported dispersal of seeds of other local myrmocochore species (both published and unpublished)** |
| --- | --- | --- | --- | --- |
| *Messor ebeninus* | Granivore | 6-13 | Social, Trunk trail formation | *Iris atropurpurea*  *Vagaria parviflora*  (Galil 1952) |
| *Messor semirufus* | Granivore | 6-13 | Social, Trunk trail formation | *Sylibum marianum* (Danin & Yom-Tov 1990)  *Sternbergia clusiana* (Shmida & Fragman-Sapir 2015) |
| *Messor arenarius* | Granivore | 5-15, Mostly 10-15 | Solitary with occasional recruitment | *none* |
| *Cataglyphis savignyi/ israelensis* | Scavenger | ≥15 | Solitary | *Vagaria parviflora*  (Galil 1952) |
| *Cataglyphis albicans* | Scavenger | ≤6 | Solitary | none |

**1.2. Interaction indices:**

We used an ordinal behavioral index that corresponds to the success of seed removal in an ant-seed interaction. We followed the classification of Culver & Beattie (1978).

**Table 1.2.1:** Interaction indices and their marks:

| **Interaction index** | **Explanation** | **Mark** |
| --- | --- | --- |
| Ignore, touch and leave | Making a contact with the seed but leaving it immediately | 1 |
| Antennate | Making a prolonged contact of the feelers with the seed | 2 |
| Examine/Elaiosome robbery | Touching the elaiosome with the mandibles or eating the elaiosome without trying to remove the seed | 3 |
| Pick up attempt | Trying to remove the seed and failing | 4 |
| Removal | Successful removal | 5 |

**1.3. Elaiosome traits – fatty acid analysis:**

In order to quantify investment in ant-rewarding traits, we analyzed elaiosomes for fatty acid composition.

Lipid extraction: To extract neutral lipids, we followed the protocol of Pal et al. (2011): lyophilized biomass samples (minimum 10 mg) were broken in a mini-bead beater (BioSpec Products) using glass beads (2 mm diameter) for 1 minute in the presence of 0.1 ml n-hexane. Six ml of n-hexane were then added, and the mixture was extracted at 60°C for 1 hour with continuous mixing under argon atmosphere. The extract was centrifuged for 5 minutes at 3,000 rpm, and the supernatant was collected. The extraction was repeated three times to get all of the neutral lipids, as confirmed by thin-layer chromatography (TLC). Total n-hexane extraction yields were determined gravimetrically: the extract was filtered through cotton wool and a 13-mm Teflon filter, and the filtrate was evaporated under nitrogen gas flow to constant weight.

Lipid separation: Neutral lipid extracts were resolved into individual classes by TLC (Silica Gel 60, 10×10 cm, 0.25 mm thickness, Merck, Darmstadt, Germany) in the solvent mixture petroleum ether: diethyl ether: acetic acid (70:30:1, v/v). Plates were briefly sprayed with iodine vapors, bands corresponding to TAG were scraped off the plates, and fatty acid profile and content were determined as fatty acid methyl esters (FAME) by capillary gas chromatography (GC). TAG were also extracted with chloroform:methanol (2:1, v/v) for analysis of molecular species. Analysis of fatty acid composition and content by GC Transmethylation of fatty acids was performed by incubating freeze-dried biomass or lipid extracts in dry methanol containing 2% (v/v) H2SO4 at 80°C for 1.5 hours under argon atmosphere and continuous stirring. Heptadecanoic acid (C17:0; Sigma–Aldrich) was added as an internal standard. FAME were quantified and qualified on Trace GC ultra (Thermo, Milan, Italy) equipped with SUPELCOWAX 10 (30 m×0.32 mm, 0.25 μm) capillary GC column (Sigma-Aldrich), a flame ionization detector and a programmed temperature vaporizing (PTV) injector. The detector temperature was fixed at 280°C, and helium was used as a carrier gas. The PTV injector was programmed to increase the temperature from 40°C at time of injection to 300°C at time of sample transfer. Separation was achieved on a fused silica capillary column (ZB-WAX+, Phenomenex, 30 m×0.32 mm). FAME were identified by co-chromatography with authentic standards (Sigma–Aldrich).

The fatty acid extraction analysis process was conducted at The French Associates Institute for Agriculture and Biotechnology of Drylands, Sde Boker, under the supervision of Inna Khozin-Goldberg and Shoshana Didi.

- 1. **Statistical analysis**

Assessing *S. clusiana* dispersal effectiveness – seed removal in cafeteria experiments

As on measure of dispersal effectiveness we modeled seed removal rates in the cafeteria experiments as a function of: geographic position with respect to *S. clusiana* range margin, ant guild, distance from a nest (distance) and within-site location (center or boundary of the local *S. clusiana* population). Because the nature of the data distribution was bi-modal, with many observation sessions summing to either zero or to complete seed removal, our first task was to choose the most suitable distribution to describe such data. The relatively large number of zeros in the data and the two-step process that may have generated the data (detection of a depot with some probability and then seed removal once detection occurred) lead us to explore a few mixture models. The distribution of such data structure can be described by a beta-binomial distribution, so that the probability $r_{\nu}$of a *S. clusiana* seed to be removed from a depot during observation ν, is described by:

$$\Pr\left( r_{\nu} | S_{\nu},\bar{P_{\upsilon}},\theta\right)=\mathrm{betabin} \left( r_{\nu} | S_{\nu},\bar{P_{\upsilon}},\theta\right)=\int_{P_{v}=0}^{1} beta\left( P_{\upsilon} | \bar{P_{\upsilon}},\theta\right)bin\left( r_{\nu} | S_{\nu},P_{\upsilon} \right)dP_{\upsilon}$$

, where $S_{\nu}$ is the number of seeds in the depot immediately before observation ν, $\bar{P_{\upsilon}}$ is the average seed detection probability and $\theta$ is a positive parameter that characterizes the variance of $\bar{P_{\upsilon}}$.

Following Bolker (2008), we used ΔAIC to compare several potential distribution structures for our data set, including the zero-inflated variance of the binomial distribution, a beta-binomial distribution and a zero-inflated beta binomial distribution (Table 1.5.1).

**Table 1.4.1:** ΔAICc values for the three distribution structures compared.

| **type of model** | **∆AICc** |
| --- | --- |
| beta binomial | 0.00 |
| zero-inflated beta-binomial | 2.00 |
| zero inflated binomial | 641.30 |

The comparison of the distribution structure of the various models suggested that the beta-binomial model is most suitable for our specific data set.

Because the outcome of the statistical inference may change in accordance with the parametrization of the beta-binomial distribution, we further tested, using ΔAICc as information criteria, the most suitable parameter combination ($\bar{P_{\upsilon}}$,$\theta)$ for each of the main model factors (site, ant guild identity of the adjacent nest, seed depot’s distance from a nest and within-site location (Table 1.5.2).

**Table 1.4.2:** A comparison between the four optional parameter combinations of the beta-binomial distribution for each of the main model factors: 1. $\bar{P_{\upsilon}}$ and$\theta$ do not change with factor’s categories (simple beta-binomial). 2. $\bar{P_{\upsilon}}$ changes with factor’s categories, $\theta$ constant ($\bar{P_{\upsilon}}$). 3. $\bar{P_{\upsilon}}$ constant, $\theta$ changes with factor’s categories ($\theta$). 4. $\bar{P_{\upsilon}}$ and$\theta$ both change with factor’s categories.

| Beta-binomial parametrization | site | ant guild | distance | location |
| --- | --- | --- | --- | --- |
| simple beta-binomial | 16.40 | 14.70 | 4.80 | 0.80 |
| $\bar{P_{\upsilon}}$ | 3.60 | 0.10 | 0.00 | 0.00 |
| $\theta$ | 15.10 | 13.40 | 6.10 | 2.80 |
| $\bar{P_{\upsilon}},\theta$ | 0.00 | 0.00 | 1.50 | 2.00 |

Overall, the results suggest that the most suitable model for our specific data set is a beta-binomial model with constant $\theta$ and $\bar{P_{\upsilon}}$ parameter change across factors (see examples in Bolker 2008). This variance was used for the factors’ combination model selection process.
