# Supplementary material for: Reduced dispersal at nonexpanding range margins: A matter of disperser identity
Source: Ecol Evol. 2020 Apr 16;10(11):4665–76. doi: 10.1002/ece3.6220 (PMC7297755; doi:10.1002/ece3.6220)
Supplement: Supplementary file 2 — Appendix S2 [file ECE3-10-4665-s002.docx]

**Appendix S2 – results complementary:**

**2.1. Assessing *S. clusiana* dispersal effectiveness – Seed removal rate:**

We chose the most parsimonious model that describes the probability of *S. clusiana* seed removal using the AICc as a model selection criteria. For each model, ΔAICc values are presented in comparison to the model with the lowest AICc.

The factors (here and hereafter) are: Site – position along a geographic gradient from the arid south next to the range margin (S1, S2), central range (C1,C2) and Northern sites (N1, N2); Guild – the ant guild occupying the focal next around which cafeteria experiments or baits were placed (Scavenger or Granivore); Distance – Distance from the focal nest (0m or 10 m for cafeteria experiments, 0m, 2.5m, 5m, 7.5m and 10m for the baits); Location – location **within** site, either at the center of *S. clusiana* population or at its edge.

**Table 2.1.1:** ΔAICc and degrees of freedom for all models relevant for the seed removal rate in the cafeteria experiments. The most parsimonious model is indicated in bold.

| **Model factors** | **ΔAICc** | **df** |
| --- | --- | --- |
| site, location, guild, distance | 0 | 10 |
| **site, guild, distance** | **1.6** | **9** |
| site, location, guild, distance, guild*distance | 1.7 | 13 |
| site, guild, distance, guild*distance | 1.9 | 12 |
| site, location, guild, distance, guild*location | 5.5 | 13 |
| site, location, guild, distance, site*guild | 7.9 | 21 |
| site, guild | 8.7 | 8 |
| site, location, guild | 9.3 | 9 |
| site, location, guild, distance, site*location | 9.7 | 21 |
| site, location, guild, distance, site*location, guild*distance | 10.4 | 24 |
| guild, distance, guild*distance | 10.8 | 7 |
| site, location, guild, distance, distance*location | 11.5 | 13 |
| guild, distance, location, guild*distance | 12.8 | 8 |
| site, location, guild, distance, site*guild, guild*distance | 13.3 | 24 |
| guild, distance | 13.6 | 4 |
| site, distance, location | 13.6 | 9 |
| site, location, guild, distance, site*location, location*guild | 13.6 | 24 |
| site, location, guild, site*guild | 13.8 | 20 |
| site, location, guild, distance, site*distance | 13.8 | 21 |
| site, location, guild, distance, site*location, location*distance | 14.1 | 24 |
| site, location, guild, site*location | 14.4 | 20 |
| site, location, guild, location*guild | 14.6 | 12 |
| site, location, guild, distance, location*guild, guild*distance | 14.6 | 16 |
| site, guild, distance, site*distance | 15.2 | 20 |
| site, location, guild, distance, site*guild, location*guild | 15.2 | 24 |
| guild, distance, location, location*distance, guild*location | 15.3 | 11 |
| guild, distance, location | 15.5 | 5 |
| site, guild, distance, guild*distance, distance*site | 15.5 | 23 |
| site, location, guild, location*guild, site*guild | 15.6 | 23 |
| site, location, guild, distance, site*location, site*guild | 15.6 | 32 |
| site, location, guild, location*guild, site*location | 15.9 | 23 |
| site, location, guild, distance, site*location, site*distance | 15.9 | 32 |
| guild, distance, location, guild*distance, distance*location | 16.2 | 11 |
| site, location, guild, distance, site*guild, location*distance | 17 | 24 |
| site, location, guild, site*location, site*guild | 17.2 | 31 |
| guild, distance, location, location*guild, distance*guild | 17.3 | 11 |
| guild, distance, location, guild*location | 17.4 | 8 |
| site, location, guild, distance, site*distance, guild*distance | 17.4 | 24 |
| guild, distance, location, guild*distance, distance*location, guild*location | 17.5 | 14 |
| site, location, guild, distance, site*distance, location*guild | 17.8 | 24 |
| site, guild, distance, guild*distance, guild*site | 18 | 23 |
| site, location, guild, distance, site*guild, site*distance | 18 | 32 |
| site, location | 18.5 | 8 |
| site, distance | 19 | 8 |
| site, guild, distance, site*guild | 19 | 20 |
| site, distance, location, site*location | 19.2 | 12 |
| site, location, guild, distance, location*distance, guild*distance | 19.6 | 16 |
| site, location, guild, distance, location*distance, location*guild | 20.1 | 16 |
| site, location, guild, distance, site*distance, location*distance | 20.4 | 24 |
| guild, distance, location, distance*location | 20.7 | 8 |
| guild | 21.5 | 3 |
| site, location, distance, location*distance | 21.8 | 12 |
| guild, distance, location, distance*location, guild*location | 22 | 11 |
| site | 23.2 | 7 |
| guild, location | 23.3 | 4 |
| guild, location, guild*location | 24.4 | 7 |
| guild, site, guild*site | 25.9 | 19 |
| site, distance, location, site*distance | 27.5 | 20 |
| Distance | 31.2 | 3 |
| distance, location | 31.8 | 4 |
| site, guild, distance, site*guild, site*distance, guild*distance | 31.8 | 34 |
| site, guild, distance, site*distance, guild*site | 32.3 | 31 |
| site, distance, site*distance | 32.5 | 19 |
| site, location, distance, site*location, location*distance | 32.9 | 23 |
| site, distance, location, site*distance, distance*location | 33.2 | 23 |
| site, location, guild, distance, site*location, site*guild, location*distance | 33.8 | 35 |
| site, location, distance, site*location, site*distance | 34.1 | 31 |
| site, location, guild, site*location, site*guild, location*guild | 34.4 | 34 |
| site, location, guild, distance, site*location, site*guild, site*distance, location*guild, location*distance, guild*distance | 35.3 | 52 |
| site, location, site*location | 35.6 | 19 |
| no-factors beta-binomial | 36.1 | 2 |
| site, location, distance, site*location, site*distance, distance*location | 36.7 | 34 |
| Location | 36.8 | 3 |
| location, distance, location*distance | 37.1 | 7 |

The model comparison suggests that the most parsimonious factor combination to describe seed removal rate in the cafeteria experiments was constructed by the additive effects of site, ant guild and distance of depot from ant nests (Table 2.1.2).

**Table 2.1.2:** Estimates (SE) of coefficients of the factors (and factor levels) that were included in the most parsimonious model affecting seed removal rate per seed depot. (Here and in all tables below, significance values are p<0.05 ***, p<0.01**, p<0.001*)

|  | Β(SE) | z value |
| --- | --- | --- |
| Intercept | 0.56(0.08) | 7.18*** |
| Site – S2 | -0.02(0.08) | -0.25 |
| Site – C1 | 0.15(0.10) | 1.58 |
| Site – C2 | 0.34(0.09) | 3.81*** |
| Site – N1 | 0.27(0.10) | 2.68** |
| Site – N2 | 0.25(0.11) | 2.40* |
| Guild - Granivore | -0.27(0.06) | -4.59*** |
| Distance - 10 meter | -0.17(0.06) | -3.06** |
| $\theta$ | 0.91(0.14) | 6.41*** |

**Table 2.1.3:** Results of Wald tests for each of the factors included in the selected model. Comparisons are between that selected model and models where one of the factors was excluded.

| **Model factors** | ΔAICc | **df** | **deviance** | **χ2** | **p(χ2)** |
| --- | --- | --- | --- | --- | --- |
| site, guild, distance (full model) | 0 | 9 | 570.71 |  |  |
| site, guild (distance effect) | 7.1 | 7 | 579.85 | 9.1356 | 0.002 ** |
| guild, distance (site effect) | 12.1 | 3 | 592.81 | 22.094 | 0.0005 *** |
| site, distance (guild effect) | 17.4 | 7 | 590.16 | 19.448 | 1.034e-05 *** |

**2.2. Assessing *S. clusiana* dispersal effectiveness – seed removal ratio between ant guilds and ant-seed interaction index**

Similar to the procedure described for seed removal probability, we first selected the most parsimonious factor combination to describe the ant guild ratio of *S. clusiana* seed removal (seeds removed by scavengers divided by seeds removed by granivores) and the interaction indices for each cafeteria experiment.

**Table 2.2.1:** ΔAICc for all factor combinations relevant for the ratio of seeds removed by scavengers/granivore ants and for interaction index in the cafeteria experiments. The most parsimonious model for each variable is indicated in bold.

| Factor combination | ΔAICc seed removal guild ratio | ΔAICc interaction index |
| --- | --- | --- |
| Site | 65.98 | 126.40 |
| Location | 56.77 | 142.87 |
| Guild | 27.64 | 48.91 |
| Distance | 56.68 | 147.33 |
| site, location | 64.45 | 123.63 |
| site, location, site*location | 38.93 | 118.10 |
| site, guild | 34.34 | 7.65 |
| site, guild, site*guild | 38.46 | 9.89 |
| site, distance | 65.44 | 128.24 |
| site, distance, site*distance | 74.51 | 137.04 |
| location, guild | 29.00 | 49.66 |
| location, guild, location*guild | 30.40 | 46.57 |
| location, distance | 56.17 | 144.26 |
| location, distance, location*distance | 54.58 | 145.81 |
| guild, distance | 22.77 | 48.21 |
| guild, distance, guild*distance | 16.40 | 43.69 |
| site, location, guild | 35.31 | 8.42 |
| site, location, guild, all 2-way interactions | 11.22 | 18.94 |
| site, location, guild, site*location, site*guild | 12.54 | 16.94 |
| site, location, guild, location*guild, site*guild | 40.89 | 13.10 |
| site, location, guild, location*guild, site*location | 18.62 | 18.17 |
| site, location, guild, site*location | 16.77 | 16.22 |
| site, location, guild, site*guild | 39.42 | 11.22 |
| site, location, guild, location*guild | 36.74 | 10.36 |
| site, location, distance | 63.53 | 125.53 |
| site, location, distance, all 2-way interactions | 45.67 | 128.55 |
| site, location, distance, site*location, site*distance | 48.30 | 127.99 |
| site, location, distance, site*location, location*distance | 36.89 | 120.52 |
| site, location, distance, site*distance, location*distance | 68.65 | 134.67 |
| site, location, distance, site*distance | 72.73 | 134.07 |
| site, location, distance, location*distance | 62.00 | 126.00 |
| site, location, distance, site*location | 38.98 | 119.79 |
| location, guild, distance | 24.07 | 48.99 |
| location, guild, distance, all 2-way interactions | 17.50 | 43.40 |
| location, guild, distance, location*guild, distance*location | 21.87 | 47.81 |
| Location, guild, distance, location*guild, distance*guild | 18.88 | 41.40 |
| location, guild, distance, distance*location, distance*guild | 16.31 | 46.14 |
| location, guild, distance, location*guild | 25.40 | 46.03 |
| location, guild, distance, distance*location | 21.25 | 50.80 |
| location, guild, distance, distance*guild | 17.62 | 44.24 |
| site, location, guild, distance | 29.42 | 34.52 |
| site, location, guild, distance, all 2-way interactions | 6.41 | 18.93 |
| site, location, guild, distance, site*location, site*guild | 9.44 | 16.77 |
| site, location, guild, distance, site*location, site*distance | 13.53 | 18.24 |
| site, location, guild, distance, site*location, location*distance | 32.41 | 16.74 |
| site, location, guild, distance, site*location, location*guild | 21.60 | 17.05 |
| site, location, guild, distance, site*location, guild*distance | 9.69 | 8.52 |
| site, location, guild, distance, site*guild, site*distance | 15.46 | 14.30 |
| site, location, guild, distance, site*guild, location*distance | 8.27 | 12.25 |
| site, location, guild, distance, site*guild, location*guild | 39.07 | 12.62 |
| site, location, guild, distance, site*guild, guild*distance | 26.77 | 6.43 |
| site, location, guild, distance, site*distance, location*distance | 33.74 | 11.80 |
| site, location, guild, distance, site*distance, location*guild | 23.14 | 11.75 |
| site, location, guild, distance, site*distance, guild*distance | 29.96 | 3.00 |
| site, location, guild, distance, location*distance, location*guild | 38.61 | 10.54 |
| site, location, guild, distance, location*distance, guild*distance | 27.94 | 3.04 |
| site, location, guild, distance, location*guild, guild*distance | 26.45 | 2.83 |
| site, location, guild, distance, site*location | 20.02 | 15.17 |
| site, location, guild, distance, site*guild | 23.03 | 10.81 |
| **site, location, guild, distance, guild*distance** | 21.67 | **0.00** |
| site, location, guild, distance, site*distance | 36.93 | 9.87 |
| site, location, guild, distance, guild*location | 30.81 | 9.01 |
| site, location, guild, distance, distance*location | 26.00 | 8.65 |
| **site, location, guild, distance, site*location, site*guild, location*distance** | **0.00** | 18.17 |

**Table 2.2.2:** Coefficient estimates of the factors (and factor levels) that were included in the most parsimonious model affecting ant-guild seed removal ratio and interaction index average per seed depot.

|  | **Ant guild seed removal ratio** | | **Interaction index** | |
| --- | --- | --- | --- | --- |
|  | **Β(SE)** | $\boldsymbol{\chi}_{\boldsymbol{1}}^{\boldsymbol{2}}$ | **Β(SE)** | $\boldsymbol{\chi}_{\boldsymbol{1}}^{\boldsymbol{2}}$ |
| **Intercept** | 0.72 | 12.56*** | 0.75 | 72.06*** |
| **Site – S1** | 0.76 | 5.18* | -0.29 | 20.35*** |
| **Site – S2** | 0.65 | 4.87* | -0.40 | 32.98*** |
| **Site – C1** | 0.48 | 3.05 | -0.08 | 1.72 |
| **Site – C2** | -0.21 | 0.61 | -0.06 | 1.13 |
| **Site – N1** | -0.40 | 2.08 | 0.01 | 0.02 |
| **Location - center** | 0.68 | 8.04** |  |  |
| **Guild - scavenger** | 0.58 | 7.14** | 0.80 | 84.19*** |
| **Distance – 1M** | 0.06 | 0.24 | 0.31 | 10.61** |
| **Site – S1*Location - center** | -1.56 | 15.66*** |  |  |
| **Site – S2*Location - center** | -1.97 | 27.38** |  |  |
| **Site – C1*Location - center** | -0.29 | 0.90 |  |  |
| **Site – C2*Location - center** | -0.41 | 1.79 |  |  |
| **Site – N1*Location – center** | -0.09 | 0.08 |  |  |
| **Site – S1*Guild – scavenger** | -0.54 | 2.00 |  |  |
| **Site – S2*Guild – scavenger** | -0.53 | 2.40 |  |  |
| **Site – C1*Guild - scavenger** | -0.32 | 1.10 |  |  |
| **Site – C2*Guild - scavenger** | 0.54 | 3.11 |  |  |
| **Site – N1*Guild - scavenger** | 0.53 | 2.95 |  |  |
| **Guild - scavenger*Distance – 1M** |  |  | -0.29 | 8.02** |
| **Location – center*Distance – 1M** | -0.64 | 11.80*** |  |  |

Guild ratio: The effect of the interaction of within-population location* depot’s distance from nest was such that depots at the population’s center and at 1M distance from nest showed lower ratio (Fig. 2.2A). The effect of the interaction of site*ant guild was significant, but no distinct trend could be detected (Fig. 2.2B).

Figures:


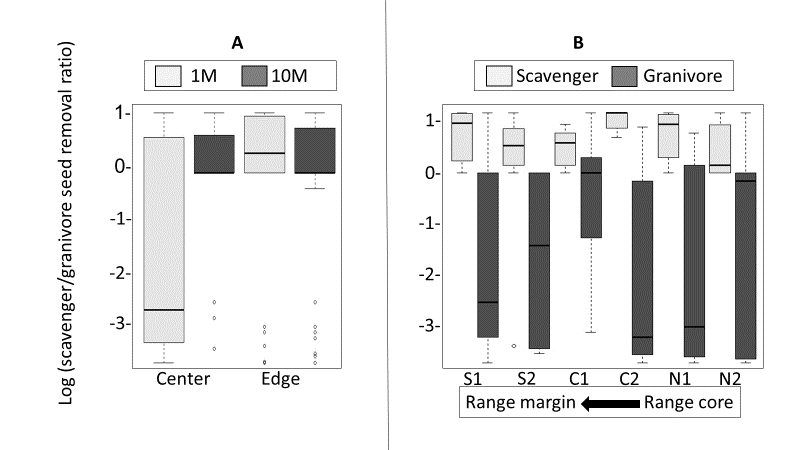


**Figure 2.1:** The effect on guild ratio of removed seeds of: **A.** Within-population location*Depot’s distance from nest. **B.** Site*Ant guild.

**2.3. Assessment of effect of diaspore traits on *S. clusiana* seed-dispersal effectiveness:**

Additional Morphological and chemical traits information:

The overall diaspore mass (mg) significantly varied between sites ($\chi_{5}^{2}=137.34, P<0.001$), with a general trend of reduction in diaspore size toward range margin (Fig. 2.3a). Similar trends were detected in the average eliaosome mass ($\chi_{5}^{2}=180.33, P<0.001$, Fig. 2.3b). Linoleic acid varied significantly between sites ($\chi_{5}^{2}=38.76, P<0.001$, Fig. 2.4a) but with no obvious trend. Much lower variation was found in the two other major fatty acids of the elaiosome: In Linolenic acid it was significant ($\chi_{5}^{2}=17.95, P=0.003$, Fig. 2.4b), in Palmitic acid non-significant ($\chi_{5}^{2}=5.38, P<0.371$, Fig. 2.4c). In both the variation did not adhere to any geographic trend; and these fatty acids, although known in many other myrmecochore species (Boulay *et al.* 2006; Fischer *et al.* 2008; Boieiro *et al.* 2012), are not related to any specific ant behavior.

**Table 2.3.1:** Estimates (SE) of coefficients of the site effect on diaspore and elaiosome traits.

|  | Morphological traits | | | | | | Chemical traits | | | | | |
| --- | --- | --- | --- | --- | --- | --- | --- | --- | --- | --- | --- | --- |
|  | Elaiosome/seed mass ratio | | Diaspore mass | | Elaiosome  mass | | Total fatty acids | | Oleic acid | | Linoleic acid | |
|  | Β(SE) | $\chi_{1}^{2}$ | Β(SE) | $\chi_{1}^{2}$ | Β(SE) | $\chi_{1}^{2}$ | Β(SE) | $\chi_{1}^{2}$ | Β(SE) | $\chi_{1}^{2}$ | Β(SE) | $\chi_{1}^{2}$ |
| Intercept | 1.03 | 1141.93*** | 150.70 | 1797.84*** | 74.64 | 2529.89*** | -0.07 | 0.30 | 31.07 | 148.21*** | 21.43 | 111*** |
| Site – S1 | -0.23 | 28.97*** | -48.09 | 91.53*** | -18.87 | 80.86*** | 0.45 | 8.11** | 0.30 | 0.01 | -8.61 | 8.96** |
| Site – S2 | -0.14 | 10.51*** | -44.36 | 79.78*** | -18.46 | 79.29*** | 0.33 | 3.95* | -1.14 | 0.10 | 0.85 | 0.09 |
| Site – C1 | 0.15 | 11.50*** | -46.56 | 85.80*** | -26.51 | 159.61*** | -0.16 | 0.63 | 2.41 | 0.45 | -2.94 | 1.05 |
| Site – C2 | 0.20 | 21.68*** | -23.01 | 20.96*** | -17.81 | 71.99*** | 0.18 | 1.03 | 8.65 | 5.74* | -4.58 | 2.54 |
| Site – N1 | -0.12 | 7.57** | -31.91 | 41.29*** | -16.50 | 35.97*** | 0.02 | 0.01 | -8.11 | 5.05* | 8.04 | 7.81** |


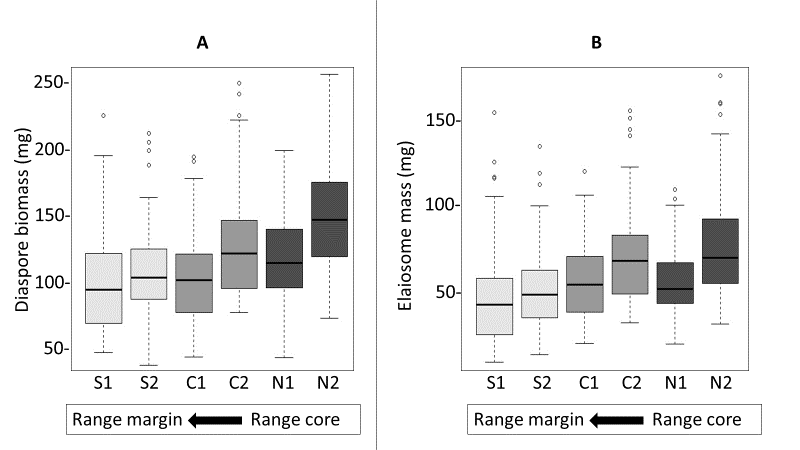


**Figure 2.2:** Variation across sites in: **A.** Diaspore mass. **B.** Elaiosome mass.


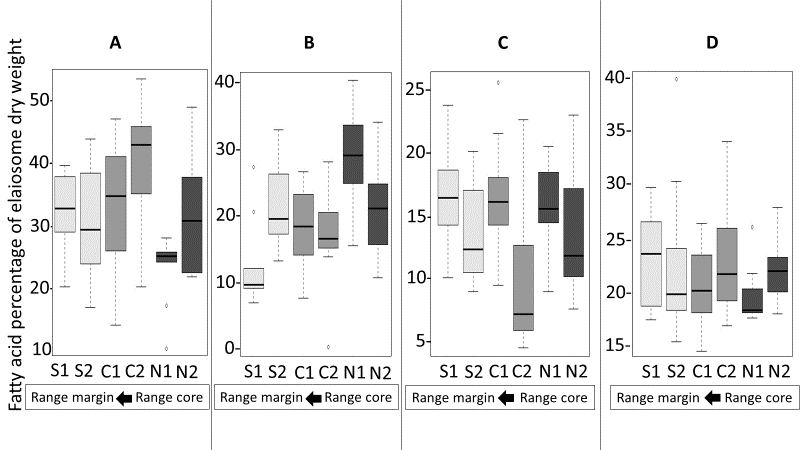


**Figure 2.3:** variation across sites in: **A.** Oleic acid. **B.** Linoleic acid. **C.** Linolenic acid. **D.** Palmitic acid.

**2.4. Assessment of the effects of ant-guild availability on *S. clusiana* seed dispersal effectiveness:**

The most parsimonious model of scavenger ants’ availability included the effects of site, Ant guild of the focal nest, location within the population, depot’s distance from the nest and the interactions of site*location, ant guild*distance and site*distance (Tables 2.4.1-2.4.3); while the most parsimonious model of granivore ants’ availability included the effects of ant guild’s nest, depot’s distance from nest, site, location within the population and the interaction of site*guild (Tables 2.4.1-2.4.2, 2.4.4).

**Table 2.4.1:** ΔAICc for all factor combinations relevant for the availability of scavengers and granivores. The most parsimonious model for each variable is indicated in bold

| Factor combination | ΔAICc number of scavengers | ΔAICc number of granivores |
| --- | --- | --- |
| site | 143.69 | 54.34 |
| location | 188.36 | 49.02 |
| guild | 182.49 | --- |
| distance | 199.35 | 13.56 |
| site, location | 89.33 | 49.93 |
| site, location, site*location | 87.95 | 51.4 |
| site, guild | 78.18 | --- |
| site, guild, site*guild | 88.17 | --- |
| site, distance | 137.83 | 8.19 |
| site, distance, site*distance | 160.62 | 53.56 |
| location, guild | 163.14 | --- |
| location, guild, location*guild | 165.09 | --- |
| location, distance | 171.85 | 9.3 |
| location, distance, location*distance | 179.89 | 17.5 |
| guild, distance | 148.07 | --- |
| guild, distance, guild*distance | 150.73 | --- |
| site, location, guild | 58.75 | --- |
| site, location, guild, all 2-way interactions | 68.77 | --- |
| site, location, guild, site*location, site*guild | 66.74 | --- |
| site, location, guild, location*guild, site*guild | 63.59 | --- |
| site, location, guild, location*guild, site*location | 63.02 | --- |
| site, location, guild, site*location | 60.89 | --- |
| site, location, guild, site*guild | 65.68 | --- |
| site, location, guild, location*guild | 57.31 | --- |
| site, location, distance | 80.03 | 2.09 |
| site, location, distance, all 2-way interactions | 98.03 | 58.93 |
| site, location, distance, site*location, site*distance | 88.21 | 47.21 |
| site, location, distance, site*location, location*distance | 83.13 | 9.08 |
| site, location, distance, site*distance, location*distance | 100.17 | 58.77 |
| site, location, distance, site*distance | 92.21 | 47.82 |
| site, location, distance, location*distance | 87.67 | 10.27 |
| **site, location, distance, site*location** | 76.75 | **0** |
| location, guild, distance | 125.58 | --- |
| location, guild, distance, all 2-way interactions | 137.18 | --- |
| location, guild, distance, location*guild, distance*location | 135.13 | --- |
| Location, guild, distance, location*guild, distance*guild | 129.36 | --- |
| location, guild, distance, distance*location, distance*guild | 135.01 | --- |
| location, guild, distance, location*guild | 127.43 | --- |
| location, guild, distance, distance*location | 133.38 | --- |
| location, guild, distance, distance*guild | 127.4 | --- |
| site, location, guild, distance | 29.59 | --- |
| site, location, guild, distance, all 2-way interactions | 13.25 | --- |
| site, location, guild, distance, site*location, site*guild | 33.83 | --- |
| site, location, guild, distance, site*location, site*distance | 17.97 | --- |
| site, location, guild, distance, site*location, location*distance | 36.82 | --- |
| site, location, guild, distance, site*location, location*guild | 33.56 | --- |
| site, location, guild, distance, site*location, guild*distance | 24.13 | --- |
| site, location, guild, distance, site*guild, site*distance | 14.42 | --- |
| site, location, guild, distance, site*guild, location*distance | 40.12 | --- |
| site, location, guild, distance, site*guild, location*guild | 31.81 | --- |
| site, location, guild, distance, site*guild, guild*distance | 25.8 | --- |
| site, location, guild, distance, site*distance, location*distance | 25.54 | --- |
| site, location, guild, distance, site*distance, location*guild | 15.62 | --- |
| site, location, guild, distance, site*distance, guild*distance | 4.56 | --- |
| site, location, guild, distance, location*distance, location*guild | 32.34 | --- |
| site, location, guild, distance, location*distance, guild*distance | 26.72 | --- |
| site, location, guild, distance, location*guild, guild*distance | 22.65 | --- |
| site, location, guild, distance, site*location | 31.48 | --- |
| site, location, guild, distance, site*guild | 34.39 | --- |
| site, location, guild, distance, guild*distance | 24.36 | --- |
| site, location, guild, distance, site*distance | 16.01 | --- |
| site, location, guild, distance, guild*location | 28.44 | --- |
| site, location, guild, distance, distance*location | 35.27 | --- |
| **site, location, guild, distance, site*distance, guild*distance, site*location** | **0** | --- |
| site, location, guild, distance, site*distance, guild*distance, site*guild | 7.76 | --- |
| site, location, guild, distance, site*distance, guild*distance, guild*location | 3.05 | --- |
| site, location, guild, distance, site*distance, guild*distance, location*distance | 12.88 | --- |

**Table 2.4.2:** Factor effect (Wald $\chi^{2}$) results of baits model for the two ant-guilds.

| Factor ($\chi^{2}\left( df \right)$) | Scavengers | Granivores |
| --- | --- | --- |
| site | 1.70(5) | 20.10(5)*** |
| location | 0.06(1) | 5.21(1)* |
| ant guild | 25.89(1)*** | Not included in model, but highly influential |
| bait distance | 8.23(4) | 60.67(4)*** |
| site*location | 5.72(5) | 11.82(5)* |
| ant guild*distance | 18.16(4)*** |  |
| site*distance | 33.29(20) |  |

**Table 2.4.3:** Estimates (SE) of coefficients of the factors (and factor levels) that were included in the most parsimonious model affecting scavengers’ number in baits.

| **Factor** | **Β(SE)** | $\boldsymbol{\chi}_{\boldsymbol{1}}^{\boldsymbol{2}}$ |
| --- | --- | --- |
| Intercept | 1.06 | 24.50*** |
| Site-S1 | -1.92 | 2.13 |
| Site-S2 | 0.59 | 5.36* |
| Site-C1 | -1.30 | 3.47 |
| Site-C2 | -1.34 | 2.70 |
| Site-N1 | -2.14 | 1.23 |
| Location-core | -0.39 | 2.73 |
| Ant guild nest -scavenger | 0.01 | 0.01 |
| Distance from nest-0 meter | -1.19 | 7.01** |
| Distance from nest-2.5 meters | -0.83 | 3.47 |
| Distance from nest-5 meters | -2.18 | 2.70 |
| Distance from nest-7.5 meters | -2.29 | 4.50* |
| Site-S1 * location-center | -1.73 | 0.02 |
| Site-S2 * location-center | -5.43 | 0.02 |
| Site-C1 * location-center | 0.26 | 0.35 |
| Site-C2 * location-center | -0.22 | 0.20 |
| Site-N1 * location-center | -0.93 | 4.21* |
| Ant guild-scavenger*Distance from nest-0 meter | 1.83 | 16.87*** |
| Ant guild-scavenger*Distance from nest-2.5 meters | 0.45 | 1.57 |
| Ant guild-scavenger*Distance from nest-5 meters | 0.47 | 2.03 |
| Ant guild-scavenger*Distance from nest-7.5 meters | 0.94 | 3.7 |
| Site-S1 * Distance from nest-0 meter | 1.13 | 0.71 |
| Site-S1 * Distance from nest-2.5 meters | 0.41 | 0.05 |
| Site-S1 * Distance from nest-5 meters | 1.32 | 0.22 |
| Site-S1 * Distance from nest-7.5 meters | 0.85 | 0.08 |
| Site-S2 * Distance from nest-0 meter | -0.24 | 0.66 |
| Site-S2 * Distance from nest-2.5 meters | 0.30 | 0.49 |
| Site-S2 * Distance from nest-5 meters | 1.91 | 2.12 |
| Site-S2 * Distance from nest-7.5 meters | 1.41 | 1.90 |
| Site-C1 * Distance from nest-0 meter | 0.83 | 1.35 |
| Site-C1 * Distance from nest-2.5 meters | 0.05 | 0.002 |
| Site-C1 * Distance from nest-5 meters | 1.90 | 1.43 |
| Site-C1 * Distance from nest-7.5 meters | 1.16 | 0.55 |
| Site-C2 * Distance from nest-0 meter | 1.02 | 1.40 |
| Site-C2 * Distance from nest-2.5 meters | 0.77 | 0.50 |
| Site-C2 * Distance from nest-5 meters | 1.88 | 1.19 |
| Site-C2 * Distance from nest-7.5 meters | 1.65 | 1.22 |
| Site-N1 * Distance from nest-0 meter | 2.64 | 1.87 |
| Site-N1 * Distance from nest-2.5 meters | 1.75 | 0.73 |
| Site-N1 * Distance from nest-5 meters | 2.96 | 1.51 |
| Site-N1 * Distance from nest-7.5 meters | 1.78 | 0.43 |

**Table 2.4.4:** Estimates (SE) of coefficients of the factors (and factor levels) that were included in the most parsimonious model affecting granivores’ presence in baits.

| **Factor** | **Β(SE)** | $\boldsymbol{\chi}_{\boldsymbol{1}}^{\boldsymbol{2}}$ |
| --- | --- | --- |
| Intercept | 3.34 | 85.05*** |
| Site-S1 | 0.24 | 0.81 |
| Site-S2 | -0.16 | 0.25 |
| Site-C1 | -0.44 | 1.28 |
| Site-C2 | 0.55 | 5.17* |
| Site-N1 | 0.52 | 4.55* |
| Location-core | -0.47 | 1.42 |
| Ant guild-scavenger | 4.92 | 24.87*** |
| Distance from nest-0 meter | 1.45 | 21.83*** |
| Distance from nest-2.5 meters | 0.90 | 7.63** |
| Distance from nest-5 meters | 0.61 | 3.14 |
| Distance from nest-7.5 meters | 0.23 | 0.36 |
| Site-S1 * location-core | -0.32 | 0.34 |
| Site-S2 * location-core | 0.82 | 2.76 |
| Site-C1 * location-core | 0.67 | 1.39 |
| Site-C2 * location-core | 0.21 | 0.23 |
| Site-N1 * location-core | -0.56 | 1.07 |
